# High parasite burden increases the surfacing and mortality of the manila clam (*Ruditapes philippinarum*) in intertidal sandy mudflats on the west coast of Korea during hot summer

**DOI:** 10.1186/s13071-018-2620-3

**Published:** 2018-01-18

**Authors:** Ki-Woong Nam, Hee-Do Jeung, Jae-Hee Song, Kwan-Ha Park, Kwang-Sik Choi, Kyung-Il Park

**Affiliations:** 1 Department of Aquatic Life Medicine, College of Ocean Science and Technology, Kunsan National University, 558 Daehakno, Gunsan, 54150 Republic of Korea; 2 Tidal Flat Research InstituteNational Institute of Fisheries Sciences, 11 Seollim-gil, Gunsan, 54014 Republic of Korea; 3 School of Marine Biomedical Science, Jeju National University, 102 Jejudaehakno, Jeju, 63243 Republic of Korea

**Keywords:** *Ruditapes philippinarum*, Thermal stress, *Perkinsus olseni*, Cell-mediated immunity, Surfaced clam, Mortality

## Abstract

**Background:**

Over the past few decades, mass mortality events of Manila clams have been reported from several tidal flats on the west coast of Korea during hot summers. During such mortality events, once clams simultaneously surface, they fail to re-burrow, perishing within a week. The present study aimed to identify the possible causes of the mass mortality of this clam species by investigating the *Perkinsus olseni* parasite burden and immune parameters of surfaced clams (SC) and normal buried clams (NBCs) when sea water or sediment temperature in the study area varied from 25 °C to 34 °C from late July through mid-August 2015.

**Results:**

We collected 2 groups of clams distributed within a 10-m^2^ area when a summer clam mortality event occurred around Seonyu-do Island on the west coast of Korea in 2015. The clams were collected 2 days after they surfaced on the sediment and still looked healthy without any gaping. The clams were transported to the laboratory, and we compared *P. olseni* infection intensity and cell-mediated hemocyte parameters between the NBCs and SCs. SCs showed significantly higher levels of *P. olseni* burden, lower condition index, and lower levels of cell-mediated immune functions than those of NBCs.

**Conclusions:**

Our study suggests that high *P. olseni* infection weakens Manila clams’ resistance against thermal stress, causing them to surface. We surmise that the summer mass mortality of Manila clams on the west coast of Korea is caused by the combined effects of high *P. olseni* infection levels and abnormally high water temperature stress.

## Background

Common on intertidal sandy mudflats on the west and south coasts of Korea, the Manila clam (*Ruditapes philippinarum*) is one of the keystone aquaculture species supporting the local shellfish industries [1–3]. In particular, the west coast of Korea (i.e. coastal Yellow Sea) includes numerous well-developed tidal flats, and these habitats often serve as clam culture grounds [4–6]. For the past 2 decades, Manila clam landings in Korea have dropped, and mass mortalities of clam in early spring or summer have been ascribed to this decline [7, 8]. While the causes of clam mass mortality are presumed to be high temperature, deterioration of water quality, and disease, the exact cause has not been identified until now [8].

*Perkinsus olseni* is a protozoan parasite infecting Manila clams all along the Korean coast. According to Park & Choi [9], the prevalence of *P. olseni* in the major clam beds on the west often reaches around 100%, and the infection intensity exceeds 1.0 × 10^6^ cells/g clam tissue in some tidal flats. Park et al. [10] first demonstrated the detrimental effects of heavy infection with *P. olseni* in Manila clam on the west coast of Korea, including retarded growth and poor reproductive performance. Flye-Sainte-Marie et al. [11] reported that Manila clams heavily infected with *P. olseni* exhibited low hemocyte phagocytosis rates, suggesting that high infection burden deteriorates clam immune function.

In mid-August 2015, mass mortality of Manila clams occurred on a tidal flat on the west coast of Korea. During the mortality event, a large number of adult clams suddenly and almost simultaneously emerged on the tidal flat surface within a day, and most of them failed to re-burrow back into the sediment. The surfaced clams appeared clinically normal and did not gape for 1–2 days. They subsequently began gaping and finally perished within a week. Unlike the surfaced clams, some of the clams remained in their habitat, at a depth of 3–5 cm below the sediment surface, during the mortality event.

By comparing *P. olseni* infection levels and immune functions of the surfaced clams (SC) and normal buried clams (NBC) collected on the west coast of Korea, we aimed to gain some insights into the role of *P. olseni* in host mortality in the summer.

## Methods

**Fig. 1:**
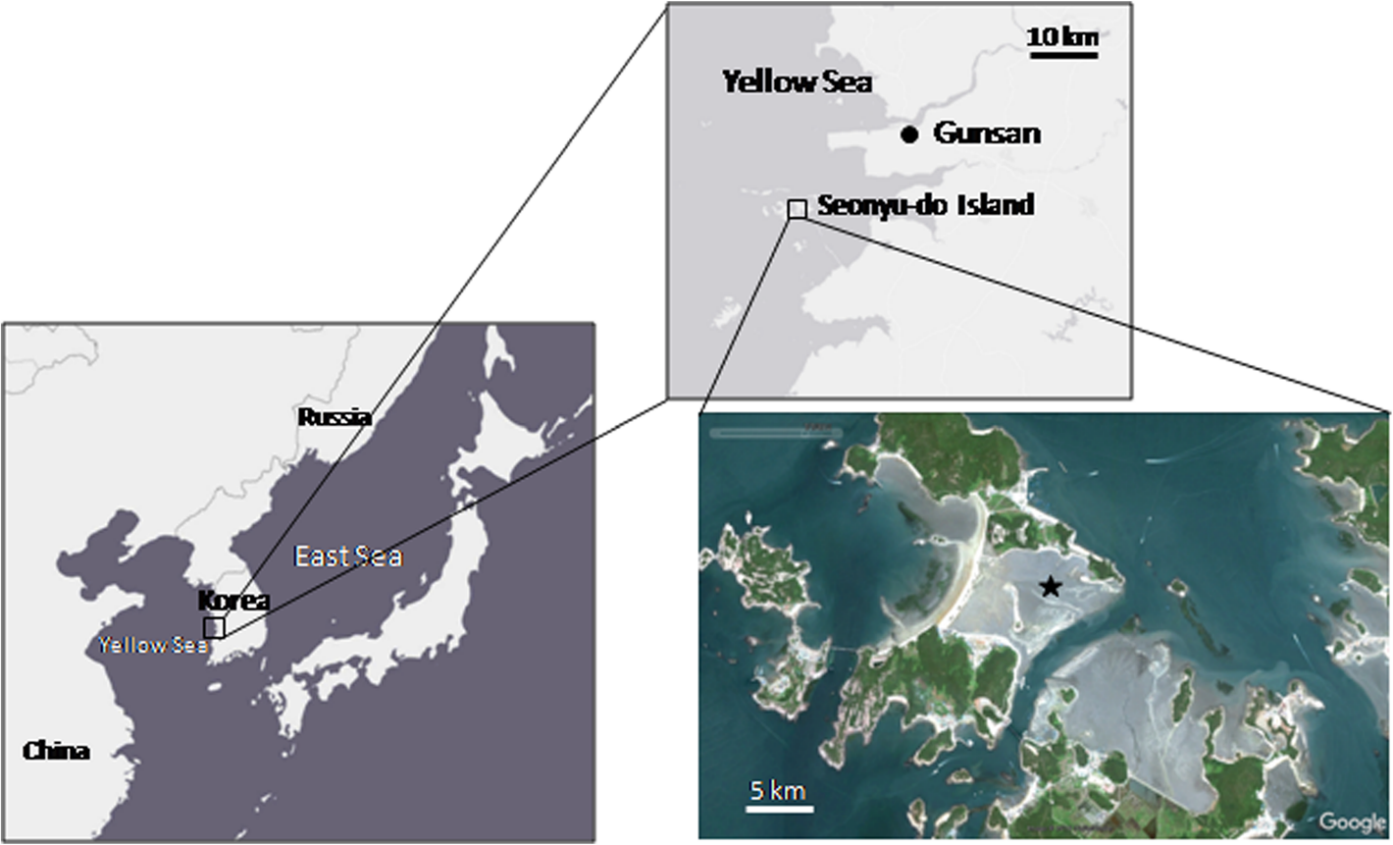
Map showing the sampling area. Clams were collected from the tidal flats on Seonyu-do Island (indicated by a star) on the west coast of Korea, when mass mortality occurred

In mid-August 2015, a mass mortality event of Manila clams occurred on the tidal flats of Seonyu-do Island on the west coast of Korea (Fig. 1). Numerous clams emerged on the sediment surface from 3 to 5 cm below the surface (i.e. SC). To determine the *P. olseni* infection level and hemocyte functions of live SC, we first monitored the emergence of clams on the sediment surface during low tide at the sampling location. On August 16, 2015, we first observed the emergence of clams on the sediment surface, and the number of emerging clams began increasing. On August 18, we collected 20 “freshly” emerged live clams (i.e. SC, 3–4 cm in shell length) during low tide for the analysis. As a control, 20 adult-sized clams located 3–5 cm underneath the sediment surface (i.e. NBC) were also collected and transported to the laboratory within 2 h. Both groups of clams were collected from the same location (within a 10 m^2^ area). In the laboratory, all clams were acclimated in an aerated seawater tank (30 psu, 25 °C) over 24 h prior to the analysis.

To evaluate immune parameters, hemolymph was harvested from each clam by inserting a 26G × 1/2″ needle into the posterior adductor muscle sinus through the shell hinge. To measure phagocytosis capacities of hemocytes, 100 μl of hemolymph was mixed with the same volume of filtered sea water. The mixed samples were mixed with 30 μl solution of fluorescent beads (2.0 μm, Polysciences Inc., Warrington, USA), which was prepared by diluting 30 μl of crude beads in 1500 μl filtered water and allowed to react for 1 h. After the completion of the incubation, the hemocyte-FITC-stained microbead solution was fixed with 1% formalin and analyzed using a flow cytometer (Gallios, Beckman Coulter, Brea, USA) and Kaluza software 1.2. On an FSC log and SSC log plot, hemocytes containing FITC-stained beads were gated and quantified on an FL1 histogram. Phagocytic activity was expressed as the percentage of phagocytic hemocytes (%), which is the ratio of the number of hemocytes that engulfed 3 or more fluorescent beads to the total number of hemocytes gated.

To determine apoptosis and necrosis rates, we used the Annexin V-FITC Apoptosis Detection Kit (BD Bioscience, San Diego, USA) according to the manufacturer’s instructions. Briefly, 100 μl of hemolymph of each clam was mixed with the same volume of FITC-conjugated Annexin V and propidium iodide (PI), incubated for 15 min, and mixed with binding buffer (0.1 M HEPES, pH 7.4; 1.4 M NaCl; 25 mM CaCl_2_). Flow cytometry was used to identify the hemocytes stained with FITC, PI, or both chemicals using Kaluza software 1.2. Accordingly, live, apoptotic, necrotic, or dead hemocytes could be identified and categorized on the basis of different types of staining determined by flow cytometry. All hemocyte assays were carried out individually.

After the completion of hemolymph withdrawal, the clams were opened, and the bodies were removed and weighed. The length and weight of the shells were recorded to determine the condition index (CI) and the ratio of tissue wet weight (TWW) to shell dry weight. The number of *P. olseni* cells in the SC and NBC used in the hemocyte function analysis was determined using Ray’s fluid thioglycollate medium (RFTM) assay [12] and Choi’s 2 M NaOH digestion technique [9, 13]. For the assay, whole clam tissue was placed in 10 ml RFTM fortified with antibiotics (penicillin and streptomycin, 10,000 units/ml, Gibco, Sparks, USA) and cultured in the dark for over 2 weeks. RFTM-incubated whole clam tissue was digested in 2 M NaOH, and the number of *P. olseni* cells in the tissue was counted using a hemocytometer under a light microscope. The level of *P. olseni* infection in each clam was expressed as the number of *P. olseni* cells/g tissue. Statistical analysis was performed using the SPSS 12.0.1 statistical package, and statistical significance was set at *P* < 0.05. Correlation analysis was performed on all parameters investigated for both groups.

## Results

Table 1 summarizes the biometry and CI of clams analyzed in this study. The mean shell length (SL) of NBC (36.59 ± 3.61 mm) and SC (38.62 ± 3.19 mm) suggested that the clams used in this analysis were 3–4-year-old adults. Shell lengths (SL) of NBC and SC were not significantly different. However, the CI of SC (0.55 ± 0.11) was significantly lower than that of NBC (0.70 ± 0.12), suggesting that the overall health condition of SC was poorer than that of NBC.

**Table 1:** Sizes and condition indices (mean ± standard deviation) of the buried and surfaced clams collected from Seonyu-do Island, Gunsan, on the west coast of Korea

Clam type	*n*	SL (mm)	Tissue wet weight (g)	CI
NBC	20	36.59 ± 3.61	3.28 ± 0.88	0.70 ± 0.12
SC	20	38.62 ± 3.19	3.28 ± 1.01	0.55 ± 0.11
*t*-test		*t*_(40)_ = -1.880, *P* = 0.068	*t*_(40)_ = -2.063, *P* = 0.054	*t*_(40)_ = -2.247, *P* = 0.037

*Abbreviations*: *NBC* normal buried clam, *SC* surfaced clam, *CI* condition index, *SL* Shell length

**Fig. 2:**
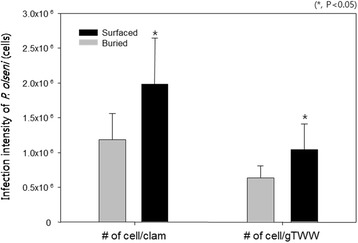
Comparison of the infection intensity of *P. olseni* between buried and surfaced Manila clams (*R. philippinarum*). *P < 0.05

The prevalence of infection with *P. olseni*, as the percentage of infected clams in the population, was 100% in both NBC and SC, indicating that all the clams used in the analysis were infected with *P. olseni*. However, the infection intensity of SC (6.63 ± 0.29 × 10^5^ cells/individual clam, or 1.98 ± 1.01 × 10^6^ cells/g TWW) was significantly higher than that of NBCs (3.72 ± 0.67 × 10^5^ cells/individual clam, or 1.16 ± 0.49 × 10^6^ cells/g TWW) (Fig. 2).

The phagocytosis rate of NBCs (18.42 ± 3.95%) was significantly higher than that of SC (11.61 ± 6.57%) (Table 2). The percentage of live hemocytes in the total hemocyte population in NBC (93.39 ± 3.55%) was also significantly higher than that of SC (75.97 ± 20.43%). Accordingly, the percentage of dead hemocytes in the total population was significantly higher in SC (6.66 ± 7.44%) than in NBC (1.58 ± 0.91%). SC also exhibited a significantly higher percentage of necrotic hemocytes (15.17 ± 12.57%), which is approximately 7-fold higher than that of NBC (2.20 ± 1.09%). However, there were no clear differences in hemocyte apoptosis between SCs and NBCs, and the apoptosis rate was approximately 2% in both clam types.

**Table 2:** Comparison of immune parameters (mean ± standard deviation) between the buried and surfaced clams

Clam type	*n*	Live cells (%)	Necrotic cells (%)	Apoptotic cells (%)	Dead cells (%)	Phagocytosis rate (%)
NBC	20	93.39 ± 3.55	2.20 ± 1.09	2.84 ± 1.99	1.58 ± 0.91	18.42 ± 3.95
SC	20	75.97 ± 20.43	15.17 ± 12.57	2.20 ± 2.07	6.66 ± 7.44	11.61 ± 6.57
Fisher’s exact test		*P* < 0.0001	*P* < 0.0001	*P* = 0.5826	*P* < 0.0001	*P* = 0.0003

*Abbreviations*: *NBC* normal buried clam, *SC* surfaced clam

Correlation analysis showed that *P. olseni* infection intensity was positively correlated with the percentage of necrotic and dead hemocytes in the host, whereas negative correlations were observed between parasite infection and CI and live hemocytes (Table 3).

**Table 3:** Correlation coefficients among size measurements, immune parameters, and *Perkinsus olseni* infection intensities of Manila clams. Data reflect measurements for all clams (*n* = 40)

	Shell length	Wet weight	CI	No. of *P. olseni*	Live (%)	Necrosis (%)	Apoptosis (%)	Dead (%)
Shell length	–							
Wet weight	0.72^**^	–						
CI	-0.14	0.49^*^	–					
No. of *P. olseni*	0.01	-0.45^*^	-0.62^**^	–				
Live (%)	-0.09	0.32	0.58^**^	-0.47^*^	–			
Necrosis (%)	0.14	-0.31	-0.65^**^	0.49^*^	-0.98^**^	–		
Apoptosis (%)	-0.17	-0.10	0.16	-0.14	-0.26	0.11	–	
Dead (%)	0.06	-0.32	-0.52^*^	0.50^*^	-0.97^*^	0.92^**^	0.21	–
Phagocytic index (%)	-0.18	0.18	0.45^*^	-0.29	0.83^**^	-0.81^**^	-0.40	-0.72^**^

^*^P < 0.05, ^**^P < 0.01

*Abbreviation*: *CI*, condition index

## Discussion

With regard to the effects of thermal stress on clams, a number of studies have reported a threshold temperature for clam immunity or mortality. Isono et al. [14] reported that *R. philippinarum* probably suffers from thermal stress at temperatures over 25 °C, with significant mortality at around 34 °C within a few days. Similarly, a temperature range of 15–28 °C is optimal for growth, although the species can survive at 0 °C and 35 °C for short periods of time [2]. Macho et al. [15] observed a 33% mortality rate in Manila clams after two days of tidal exposure to 36 °C, although no mortality occurred when the clams were exposed to 34 °C. These studies indicate that unusually high water temperatures cause severe physiological stress to marine bivalves.

**Fig. 3:**
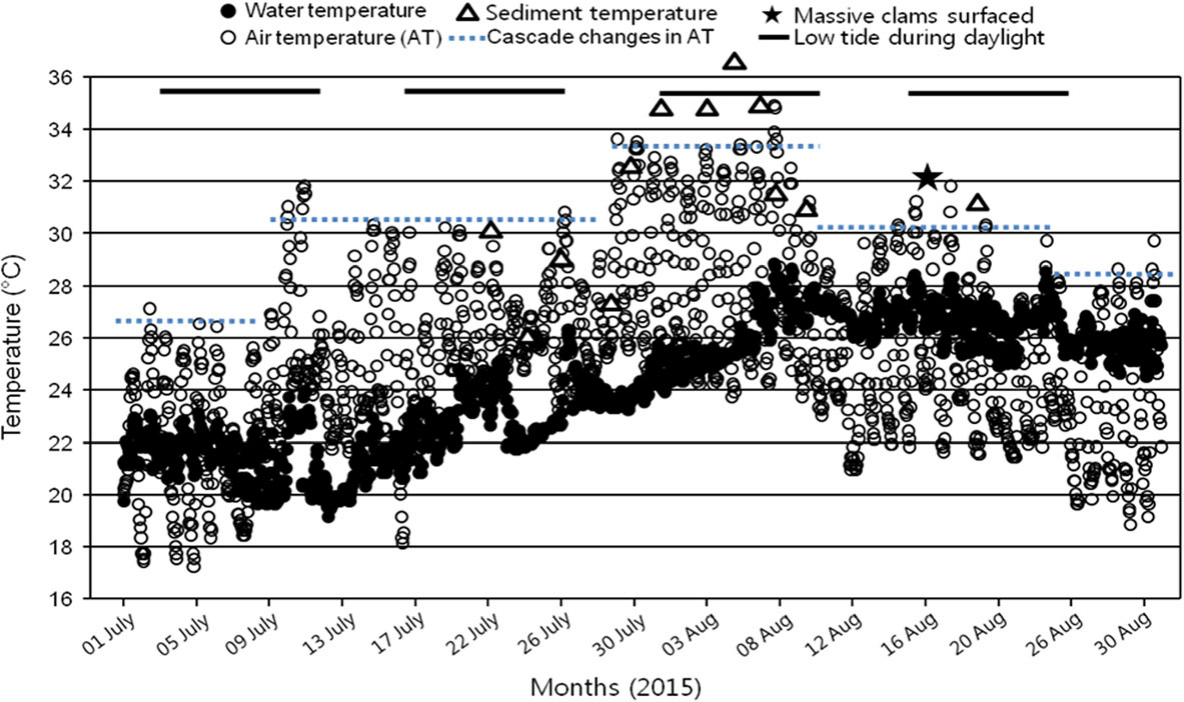
Hourly changes in sea water and air temperatures measured in July and August 2015 (the recording site is about 10 km south of the sampling site (source [16, 17])

According to the Korea Meteorological Administration [16] and Korea Hydrographic and Oceanographic Agency [17], the air temperature and surface seawater temperature around the sampling site increased rapidly in the summer of 2015. The daily maximum air temperature (DMAT) dramatically increased from 26 °C in early July to 35 °C in early August, and the highest DMAT period (over 33 °C) for the year was recorded from late July to early August for about 10 days, after which DMAT decreased to around 30 °C in mid-August. These changes in DMAT showed a cascading pattern during summer, and each step continued for about 10 days (Fig. 3). Changes in the daily maximum surface sea water temperature (DMSST) also showed the same trend as the DMAT changed, but the increase in DMSST occurred several days after the DMAT increased. DMSST increased from 23 °C in late July to 28 °C in early August in 2015, and the highest DMSST (over 27 °C) period for the year continued from mid-August to late August. During the highest DMAT period (late July to early August) in Gomso Bay, about 20 km south of the sampling site, low tide occurred during daylight, exposing the tidal flat to the hot atmosphere for 3–4 h a day (Fig. 3). The sediment temperatures varied from 32 °C to 37 °C in this period. In the present study, air, sea water and sediment temperatures of Seonyu-do Island measured on the sampling day were 30.6 °C, 26.1 °C and 31.3 °C, respectively, suggesting that the clams used in this study might have been exposed to high levels of thermal stress over a 4-week period, particularly during the low tide period in mid-July and August, before they were collected.

It is well documented that high seawater temperature deteriorates cell-mediated immune functions of marine bivalves, including total hemocyte count, phagocytosis, necrosis, and apoptosis [18, 19]. According to Gagnaire et al. [20], increased hemocyte mortality closely relates to high seawater temperature stress, which also correlates with declines in other immune functions of marine bivalves. The present study compared the physiological stress between SC and NBC by examining the rates of phagocytosis, apoptosis, necrosis, and mortality of Manila clam hemocytes, and found significant physiological disorders in SC.

Here, we quantified hemocytes that were in the process of dying, as evidenced by apoptosis and necrosis, determined by flow cytometry. Apoptosis of immune cells is involved in maintaining cellular homeostasis after exposure to various environmental and pathological stresses. According to studies on vertebrates to understand the correlation between host cell apoptosis and pathogens, intracellular parasites whose survival depends on the survival of host cells increase the host cell survival rate by inhibiting apoptosis and preventing cell death (reviewed in [21]). Similar patterns have been observed in marine bivalves. In *Crassostrea virginica* infected with *Perkinsus marinus*, apoptosis increased in the early stage of infection, but later decreased (reviewed in [22]). However, in *Crassostrea gigas*, which is more resistant to *P. marinus*, apoptosis only increased [23]. Accordingly, in our study, apoptosis in both SC and NBC might have been suppressed by *P. olseni* because all the clams used in the present study were heavily infected with the pathogen. The apoptosis rate of SC and NBC measured in the present study was about 2%, which is extremely high, considering that the apoptosis rate in healthy Manila clams reported by Park [24] was only 0.05%. The high apoptosis rate in SC and NBC could be due to other factors. Cherkasov et al. [25] reported that there was no change in the apoptosis rate in *C. virginica* hemocytes exposed to water temperatures below 25 °C, but the apoptosis rate increased under high water temperatures (> 28 °C). Therefore, it is possible that the high water temperatures in summer increased the apoptosis rate in Manila clams, thereby increasing the death rate of hemocytes.

The cell survival rate of SC in the present study was much lower than that of NBC, because the number of necrotic cells in SC was 7 times that in NBC. Little is known about factors influencing necrosis in shellfish; however, it has been reported that proteolytic enzymes released by the parasite *Perkinsus* spp. lead to necrosis (see review by Villalba et al. [26]). Therefore, one of the reasons for the high necrosis in the SC was the high level of *P. olseni* infection. Few studies have been conducted on the effects of high temperatures on necrosis in shellfish. Consequently, further studies are necessary to determine the effects of high water temperature stress on the differences in the necrosis rates between SC and NBC.

*Perkinsus* spp. are pathogens that pose a high risk to their hosts (see review by [26]). In particular, *P. olseni* brings about hemocyte infiltration or decreased immunity in the host [27–29]. It is established that high water temperature stress not only depletes metabolic energy sources in shellfish but also diminishes their ability to fight off foreign pathogens [30]. Therefore, we hypothesized that when mild thermal stress (before severe thermal stress, i.e. mid-July) persists in early summer, some clams become more susceptible to *P. olseni*, which intensifies *P. olseni* infection levels. In the present study, we also found that as *P. olseni* infection increased, the number of necrotic cells and dead cells increased in both the buried and exposed clams. Dang et al. [31] reported that an infection load of 10^5^ to 10^6^ cells of *Perkinsus* spp. can affect physiological functions in hosts. Since the SC and NBC used in the present study showed much higher infection intensities than this, the effects of *P. olseni* on clams might be much more serious. According to a report by Casas et al. [32], when *P. olseni* infection is severe, energy consumption due to *P. olseni* rises, further increasing when water temperature is high. It is also reported that high water temperature (30 °C) accelerates the trophozoite-prezoosporulation-zoosporulation cycle of *P. olseni* [33]. Accordingly, high water and sediment temperatures in summer provide favorable conditions for *P. olseni* growth by suppressing the immune response of the Manila clam, which would otherwise hinder the proliferation of *P. olseni*. We infer that a depressed immune response in summer might result in a very high infection intensity of the pathogen in Korea.

In the present study, we found that the SC showed significantly higher levels of *P. olseni* infection and lower CI than those of NBC. These phenomena were not likely to be the result of the clams being on the surface (for only two days), because the doubling time of *Perkinsus* spp. is considerably longer than the amount of time the clams spent on the surface sediment [34]. Moreover, a change in the CI in clams is not the result of instant activation of a physiological process; it is a long-term process. On the other hand, by considering the rapid immune response of bivalves to disease [35] and environmental change [36], it is highly likely that clams exposed to high sea water temperature or high air temperature during low tide in August may show dramatic immune changes. Thus, it is not clear if the significantly different immune response between SC and NBC occurred while the clams were still in the sediment or after the SC emerged. We speculate that the SC that showed higher *P. olseni* burden and lower CI were probably more fragile in their immune response than NBC when they were in the sediment together.

## Conclusions

Our findings suggest that clams from the tidal flats of Seonyu-do Island were exposed to physiological stress such as thermal stress (> 25 °C temperatures of water or sediment) during early summer, and *P. olseni* infection intensity increased in the clams during this period owing to decreased resistance to *P. olseni* from such stress. Consequently, individuals with high infection intensity and low physiological status ended up surfacing onto the tidal flats and failed to re-burrow when severe thermal stress occurred in mid-August. Thus, the recent summer mass mortality events of clams might be attributable to severe infection by *P. olseni* and the additive effects of thermal stress. Considering the continuous increase in sea water temperature in the region, it is expected that the massive decimation of the Manila clam in summer will continue in the future.
